# Phase I Clinical Trial of Fibronectin CH296-Stimulated T Cell Therapy in Patients with Advanced Cancer

**DOI:** 10.1371/journal.pone.0083786

**Published:** 2014-01-31

**Authors:** Takeshi Ishikawa, Satoshi Kokura, Tatsuji Enoki, Naoyuki Sakamoto, Tetsuya Okayama, Mitsuko Ideno, Junichi Mineno, Kazuko Uno, Naohisa Yoshida, Kazuhiro Kamada, Kazuhiro Katada, Kazuhiko Uchiyama, Osamu Handa, Tomohisa Takagi, Hideyuki Konishi, Nobuaki Yagi, Yuji Naito, Yoshito Itoh, Toshikazu Yoshikawa

**Affiliations:** 1 Department of Molecular Gastroenterology and Hepatology, Graduate School of Medical Science, Kyoto Prefectural University of Medicine, Kyoto, Japan; 2 Department of Cancer ImmunoCell Regulation, Kyoto Prefectural University of Medicine, Kyoto, Japan; 3 Center for Cell and Gene Therapy, Takara Bio Inc, Otsu, Japan; 4 Division of Basic Research, Louis Pasteur Center for Medical Research, Kyoto, Japan; Queen Elizabeth Hospital, Hong Kong

## Abstract

**Background:**

Previous studies have demonstrated that less-differentiated T cells are ideal for adoptive T cell transfer therapy (ACT) and that fibronectin CH296 (FN-CH296) together with anti-CD3 resulted in cultured cells that contain higher amounts of less-differentiated T cells. In this phase I clinical trial, we build on these prior results by assessing the safety and efficacy of FN-CH296 stimulated T cell therapy in patients with advanced cancer.

**Methods:**

Patients underwent fibronectin CH296-stimulated T cell therapy up to six times every two weeks and the safety and antitumor activity of the ACT were assessed. In order to determine immune function, whole blood cytokine levels and the number of peripheral regulatory T cells were analyzed prior to ACT and during the follow up.

**Results:**

Transferred cells contained numerous less-differentiated T cells greatly represented by CD27+CD45RA+ or CD28+CD45RA+ cell, which accounted for approximately 65% and 70% of the total, respectively. No ACT related severe or unexpected toxicities were observed. The response rate among patients was 22.2% and the disease control rate was 66.7%.

**Conclusions:**

The results obtained in this phase I trial, indicate that FN-CH296 stimulated T cell therapy was very well tolerated with a level of efficacy that is quite promising. We also surmise that expanding T cell using CH296 is a method that can be applied to other T- cell-based therapies.

**Trial Registration:**

UMIN UMIN000001835

## Introduction

Adoptive T cell transfer (ACT) is currently one of the few immunotherapies that can induce objective clinical responses in a significant number of patients with metastatic solid tumors [1]. The intrinsic properties of the ACT population, particularly its state of differentiation, are said to be crucial to the success of ACT-based approaches [2]–[5]. Less differentiated T cells have a higher proliferative potential and are less prone to apoptosis than more differentiated cells. Less differentiated T cells express receptors such as the IL-7 receptor α-chain (IL-7Rα), therefore these cells have the potential to proliferate and become fully activated in response to homeostatic cytokines such as IL-7 [6]. Results from prior clinical studies demonstrated a significant correlation between tumor regression and the percentage of persistent ACT transferred cells in the peripheral blood [3], [7]. These findings suggest that the persistence and proliferative potential of transferred T cells play a role in clinical response and that less-differentiated T cells are ideal for ACT transfer therapy. Using a standard rapid expansion protocol, T cells for ACT are usually expanded with a high dose of IL-2 and CD3-specific antibody for about 2 weeks. T cells using this protocol induce progressive T cell differentiation towards a late effector state. However, although IL-2 is essential for the persistence and growth of T cell it also has undesirable qualities, such as its ability to promote the terminal differentiation of T cells [8]. As a result, the currently used procedure results in phenotypic and functional changes of T cells that make them less optimal for mediating antitumor responses in vivo. In light of this, developing new methods to obtain less differentiated T cells is crucial for improving current T-cell-based therapies so that patients can develop a long-lasting positive immune response.

It has been reported that fibronectin (FN), a major extracellular matrix protein, functions not only as an adhesion molecule but also as a signal inducer via binding to integrins expressed on T cells [9], [10]. FN acts together with anti-CD3 to induce T cell proliferation, which is thought to depend on integrin very late activation antigen-4 (VLA-4)/CS1 interactions [11], [12]. Recombinant human fibronectin fragment (FN-CH296, RetroNectin) has been widely used for retroviral gene therapy to enhance gene transfer efficiency. FN-CH296 was also reported to be able to stimulate peripheral blood T cell growth in vitro when used together with anti-CD3 and IL-2. Anti-CD3/IL-2/FN-CH296-stimulated T cells contained a higher quantity of less-differentiated T cells and in vivo persistence of these cells was significantly higher than cells stimulated by other methods [13]. These observations led us to apply FN-CH296-mediated stimulation to less differentiated phenotype T cells to generate ‘fit T cells’ [2], [14] which are ideal for ACT. In this way, we proceeded to evaluate the safety and efficacy of FN-CH296-stimulated T cell therapy in patients with advanced cancer.

## Methods

The protocol for this trial and supporting TREND checklist are available as supporting information; see Checklist S1 and Protocol S1.

### Study Design

The clinical protocol was approved by the ethics committee of Kyoto Prefectural University of Medicine and was conducted in accordance with the Declaration of Helsinki and Ethical Guidelines for Clinical Research (the Ministry of Health, Labor and Welfare, Japan). The primary objective of this phase I clinical trial was to assess the safety and adverse-event profiles of FN-CH296-stimulated T cell therapy in patients with advanced cancer. Our secondary objective was to assess the antitumor activity of ACT therapy. This study was conducted as a standard 3+3 phase I design that investigated the dose limiting toxicities (DLTs) occurring over a 28-day period after the second infusion of cultured lymphocytes. DLT was defined as grade ≥3 for any adverse event related to the infusion of cultured cells. We used an accelerated titration design to assess the safety of the number of adoptive lymphocytes at 1×10^9^ (cohort 1), 3×10^9^(cohort 2), and 9×10^9^ (cohort 3) per person. If no DLTs were observed in the first cohort of patients, a second cohort of 3 patients were treated at the next higher dose. If DLT was observed in at least one patient, the cohort was expanded to 6 patients. If ≥2 DLTs were noted in the initial or expanded cohort, no further dose escalations were performed and the maximally tolerated dose (MTD) was considered to have been exceeded. There was no intra-patient dose escalation in this study.

Written informed consent was obtained from all patients. This study is registered in the UMIN Clinical Trials Registry with the identifier UMIN000001835.

### Patients

Patients enrolled in this trial fulfilled the following eligibility criteria: histologically or cytologically confirmed esophageal cancer, gastric cancer, colorectal cancer, pancreatic cancer, biliary tract cancer or non-small lung cancer; residual disease after standard treatment with no other curative treatment options available; no plans to receive chemotherapy other than oral fluorouracil prodrugs, radiation therapy or biological response modifiers; between 20 to 80 years of age; an Eastern Cooperative Oncology Group (ECOG) performance status of 2 or less; a life expectancy of at least three months; at least four weeks since their last chemotherapy or radiation therapy; and adequate hematologic, hepatic, renal and cardiac function.

The exclusion criteria were as follows: presence of uncontrolled infection; a history of autoimmune disease or severe hypersensitivity, presence of serious complications such as unstable angina, or myocardial infarction within six months of cancer onset, interstitial pneumonia or pulmonary fibrosis with radiological findings, presence of marked ascites or pleural effusion, ileus, active other malignancy, severe mental impairment, pregnancy or lactation, and a medical history of severe hypersensitivity.

### Preparation of the FN-CH296-stimulated T cells

Peripheral blood (50–70 mL) was taken from cancer patients. Peripheral blood mononuclear cells (PBMCs) were separated using Ficoll-Paque PREMIUM (GE Healthcare, Tokyo, Japan). Subsequently, 3–8×10^7^ cells were re-suspended in culture medium in a CultiLife215 bag (Takara Bio, Otsu, Japan) that was pre-coated with both anti-CD3 and FN-CH296 (RetroNectin®, Takara Bio), which is a recombinant fragment of human fibronectin. Cells were cultured in a serum-free medium, GT-T551 (Takara Bio), which was supplemented with 0.6–1.2% heat-inactivated autologous plasma and 200 U/mL of recombinant IL-2 (Proleukin; NovartisPharma, Nürnberg, Germany). On day 4, the cells were transferred to a CultiLife Eva bag (Takara Bio), and GT-T551 medium that was supplemented with 0.6–1.2% heat-inactivated autologous plasma and 200 U/mL of IL-2 was added. On day 7, GT-T551 medium containing 200 U/mL of IL-2 was added. On day 10, the cells were harvested and re-suspended in 50–90 mL of cryopreserved solution consisting of CP-1 (Kyokutou Seiyaku, Tokyo, Japan), RPMI1640 (KOHJIN BIO, Sakado, Japan) and human serum albumin (Albuminar; CSL Behring, PA, USA) to create the final cell product. In order to obtain the determined number of adoptive lymphocytes, the patients in cohorts 1 and 2 needed a one-time lymphocyte culture, and the patients in cohort 3 needed three lymphocyte cultures. Cultured lymphocytes were frozen and stored at −80°C until the time of transfusion.

Before infusing patients, cell products were assessed for <$>\raster(80%)="rg1"<$> viability by trypan blue exclusion assay <$>\raster(80%)="rg2"<$> for sterility by the BacT/ALERT (bioMérieux, Durham, NC, USA) microbiological detection system, and <$>\raster(80%)="rg3"<$> endotoxin by a kinetic turbidimetric LAL assay. Both sterility and endotoxin tests were contracted to FALCO Biosystems (Kyoto, Japan). Further, phenotypic markers of a small aliquot of final products were examined after the freeze-thawing. Thus, these markers are considered to be almost similar to those of the infused cells.

### Whole Blood Cytokine Assays

**Figure 1:**
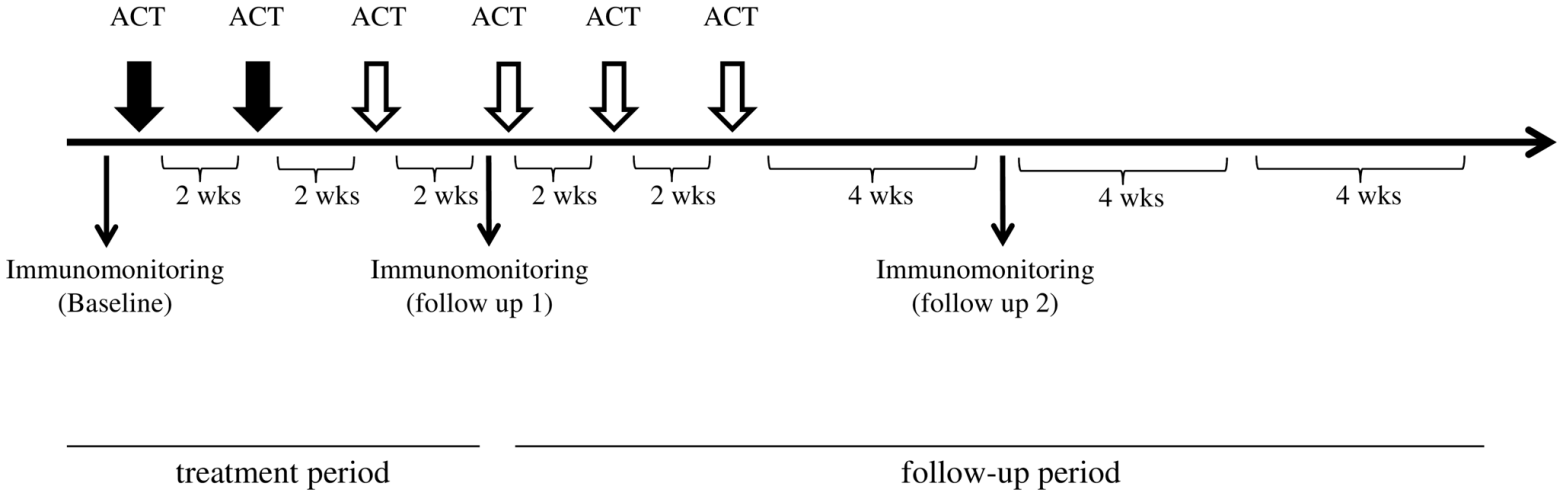
Treatment protocol. Subjects received CH296-stimulated T-cell therapy on days 1 and 15. We conducted safety evaluations for the first 6 weeks of treatment. Patients who wished to continue the treatment received up to 4 further treatments every 2 weeks. To test immune function, venous blood was obtained from subjects before the start of therapy (baseline) and during follow-up one which occurred at 4 weeks (2 treatments) and after 6 cultured cell administrations.

Immune function was tested using venous blood obtained from patients prior to administering them with cultured cells (baseline) and during the follow up which occurred 4 weeks after the 2^nd^ and 6th cultured cell infusion (Fig. 1). Methods for quantifying IFN-α production in whole human blood have been described previously [15]. Briefly, heparinized peripheral blood was cultured with Sendai virus (500 HA/mL) within 8 h after the blood sample was taken. The blood–virus mixture was incubated at 37°C for 20 h, and IFN-α activity in the supernatants was quantified by a bioassay. Other cytokines were quantified according to procedures described previously [16], [17]. Heparinized whole blood was diluted 4-fold with Eagle’s minimal essential medium (Nissui Pharmaceutical Co. Ltd., Tokyo, Japan) and stimulated with phytohemagglutinin-P (PHA-P, 25 µg/mL, Wako Pure Chemical Ind., Osaka, Japan). Samples were incubated at 37°C for 48 h, after which the supernatants were harvested by centrifugation at 800×*g* for 10 min, and then stored at −80°C until they were required for analysis. Cytokine levels in the samples were measured with a Bio-Plex multiplex cytokine array system (; Bio-Rad Laboratories, CA, USA) according to the manufacturer’s instructions. The Multiplex Th1/Th2 bead kit (Bio-Rad Laboratories) measured the following cytokines: IL-2, IL-4, IL-5, IL-10, IL-12(p70), IL-13, Tumor Necrosis Factor (TNF)-α, IFN-γ, and granulocyte–monocyte colony-stimulating factor (GM-CSF). Data acquisition and analysis were conducted with the Bio-Plex Manager Software, version 5.0. Since the levels of cytokines without PHA-P-stimulation were extremely low (data not shown), we only assessed those with PHA-P-stimulation in this study.

### Flow Cytometric Analysis

The cells were stained with FITC-, phycoerythrin (PE)-, Phycoerythrin-TexasRed (ECD)-, or Phycoerythrin-Cyanin (PC5)-conjugated mAb specific for CD3, CD4, CD8, CD27, CD45RA, CD56, HLA-DR (Beckman Coulter, Marseille, France), CD28, NKG2D (eBioscience, CA, USA) and CCR7 (R&D systems, MN, USA). To determine the regulatory T cell (Treg), we used the following antibodies: PE-Cy™5 mouse anti-human CD4, PE mouse anti-human CD25, and Alexa Fluor® 488 mouse anti-human Foxp3 (BD Biosciences Pharmingen, CA, USA). Cells were incubated at 5°C for 30 min, then washed with phosphate-buffered saline and analyzed by Cytomics FC500 (Beckman Coulter). For Foxp3 detection, cells were permeabilized overnight in Fix/Perm buffer (eBioscience) and then stained with anti-Foxp3. Data acquisition and analysis were conducted with the CXP Software, version 2.2 (Beckman Coulter). The flow data was analyzed by the following method: lymphocyte gates were determined visually in FSC/SSC dot plot after which gates with a positive region of CD3, HLA-DR were determined by staining cells with isotype control antibodies. The others (CD4, CD8, CD28, CD45RA, CD56, CD28, NKG2D and CCR7) were determined visually at a valley point of histogram or dot plot. Tregs phenotype was defined as CD4+ CD25+Foxp3+ cell.

### Study Treatment

Frozen cells were rapidly thawed at 37°C in a water bath with gentle agitation. Patients were infused intravenously for 30 min on an outpatient basis at Kyoto Prefectural University Hospital. Patients were monitored for acute toxic effects for at least one hour after infusion before they were discharged. Cultured lymphocytes were administered on days 1 and 15, and we conducted immune-monitoring to evaluate the safety of the treatment 4 weeks after the 2nd adoptive cell transfer was done. Two transfers are usually sufficient to assess the safety of novel ACT however since further treatments can be beneficial, patients could receive up to four more transfers every two weeks unless they had unacceptable toxic effects, disease progression, or withdrew consent (Fig. 1).

### Toxicity and Efficacy Assessment

Safety and toxicity was determined based on regular patient interviews, laboratory tests and physical examinations. Toxic effects were monitored according to the National Cancer Institute Common Toxicity Criteria (NCI-CTC VERSION 3.0). The relationship between adoptive cell transfer and toxicity was evaluated based on the characteristics of ACT side effects compared with those of oral fluorouracil prodrugs.

Antitumor activity was evaluated based on actual tumor response. For efficacy assessment, computed tomography (CT) was performed before the start of ACT treatment, four weeks after the 2^nd^ infusion of cultured lymphocytes and thereafter, every 4 weeks for three months. Responses were defined according to the Response Evaluation Criteria in Solid Tumors (RECIST VERSION 1.0) criteria.

### Statistical Analysis

A Wilcoxon signed ranks test was used to compare the results before and after treatment. Spearman correlation coefficient method was used to assess a possible linear association between two continuous variables. P values less than 0.05 were considered significant. All statistical analyses were performed with SPSS software (version 20) for Windows (IBM Corporation, Illinois, U.S.A.).

## Results

### Patient Characteristics

From May 2009 to December 2009, ten cancer patients were enrolled in this study. One patient discontinued due to a rapid worsening of his general condition before starting treatment. Thus nine patients were eligible for the study and assigned to three treatment cohorts. The patients’ clinical features are listed in Table 1. The median age was 60 years (range 43–75), and all patients had an ECOG performance score of 0 or 1. Three patients had colorectal cancer, two had bile duct cancer, and one patient each had pancreatic cancer, gastric cancer, hepatocellular carcinoma, and lung cancer. Of the nine patients, seven had received S-1 (an oral fluoropyrimidine) monotherapy that was combined with ACT therapy. All patients underwent two infusions of cultured cells, and eight patients who wished to continue treatment completed a further 4 T-cell infusions. Of nine patients, one (Patient no. 9) did not wish to continue the treatment, so he received two infusions of cultured cells in total.

**Table 1:** Patient characteristics.

No.	Cohort	Age (years)	Gender	Disease	ECOG/PS	Prior treatment	Combined treatment (chemotherapy)
1	Cohort 1	52	M	colonic cancer	0	1st line, mFOLFOX62nd line, S-1	S-1
2	Cohort 1	62	M	pancreatic cancer	1	1st line, GEM2nd line, S-1	S-1
3	Cohort 1	60	M	gastric cancer	0	1st line, S-1	S-1
4	Cohort 2	72	F	rectal cancer	0	1st line, mFOLFOX62nd line, Capectabine	S-1
5	Cohort 2	61	F	bile duct cancer	1	1st line, GEM2nd line, S-1	S-1
6	Cohort 2	75	M	lung cancer	1	1st line, GEM/CDDP2nd line, DTX	none
7	Cohort 3	48	M	hepatocellular carcinoma	1	1st line, 5-FU/CDDP	none
8	Cohort 3	43	M	bile duct cancer	1	1st line, GEM2nd line, GEM/S-1	S-1
9	Cohort 3	53	M	rectal cancer	1	1st line, mFOLFOX6+bevacizumab2nd line, FOLFIRI 3rd line, CPT-11+cetuximab	S-1

ECOG = Eastern Cooperative Oncology Group.

PS = performance status.

### Phenotype of Transferred Cells

**Figure 2:**
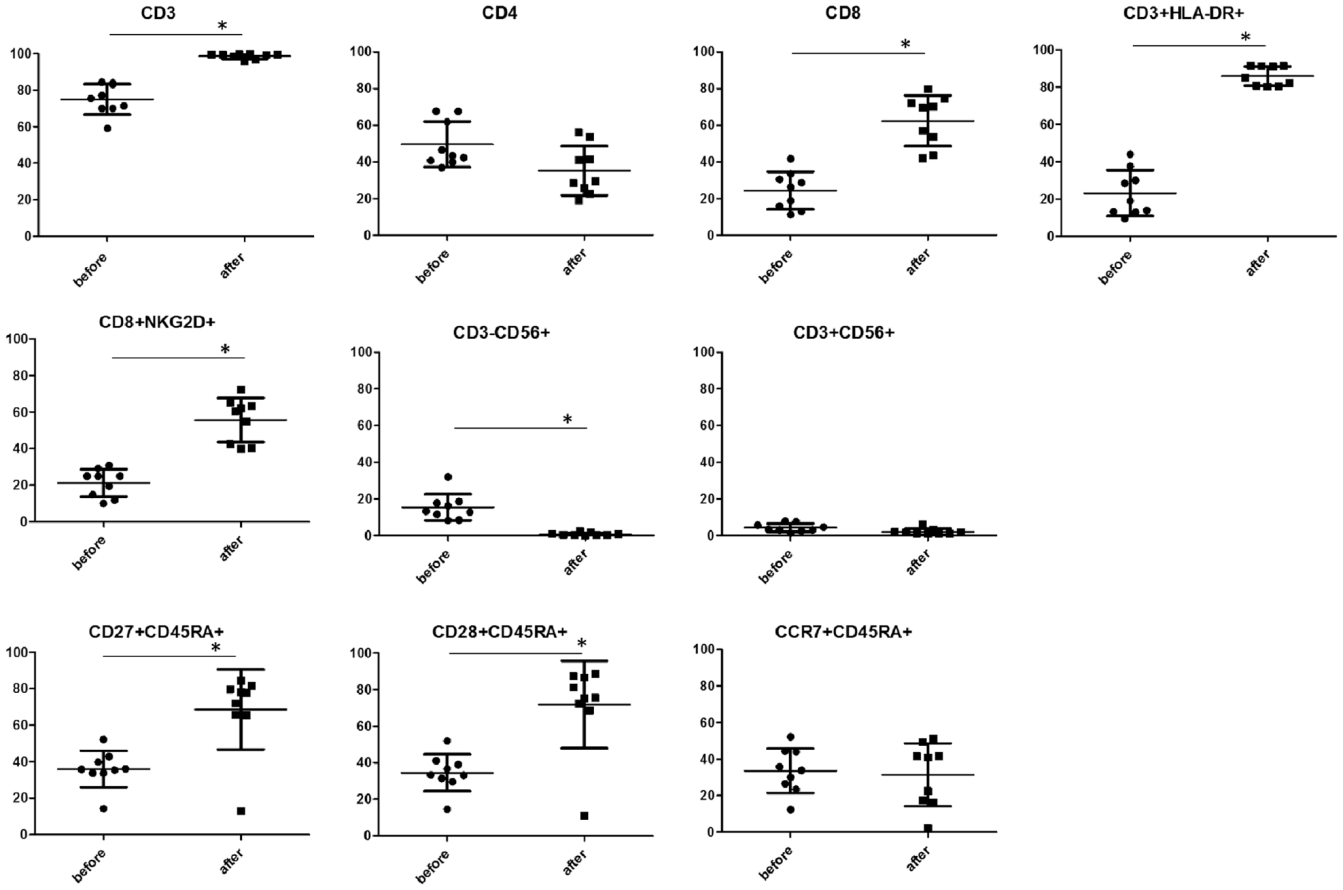
Changes in cell-surface phenotype after culture. PBMCs were stimulated with anti-CD3/CH-296. On day 10, cultured cells were harvested for transfusion. Cell-surface phenotypes of PBMCs or cultured cells were analyzed by flow cytometry. The average results from nine subjects are shown. In all panels, the lines represent the mean or standard deviation. *P<0.05.

**Figure 3:**
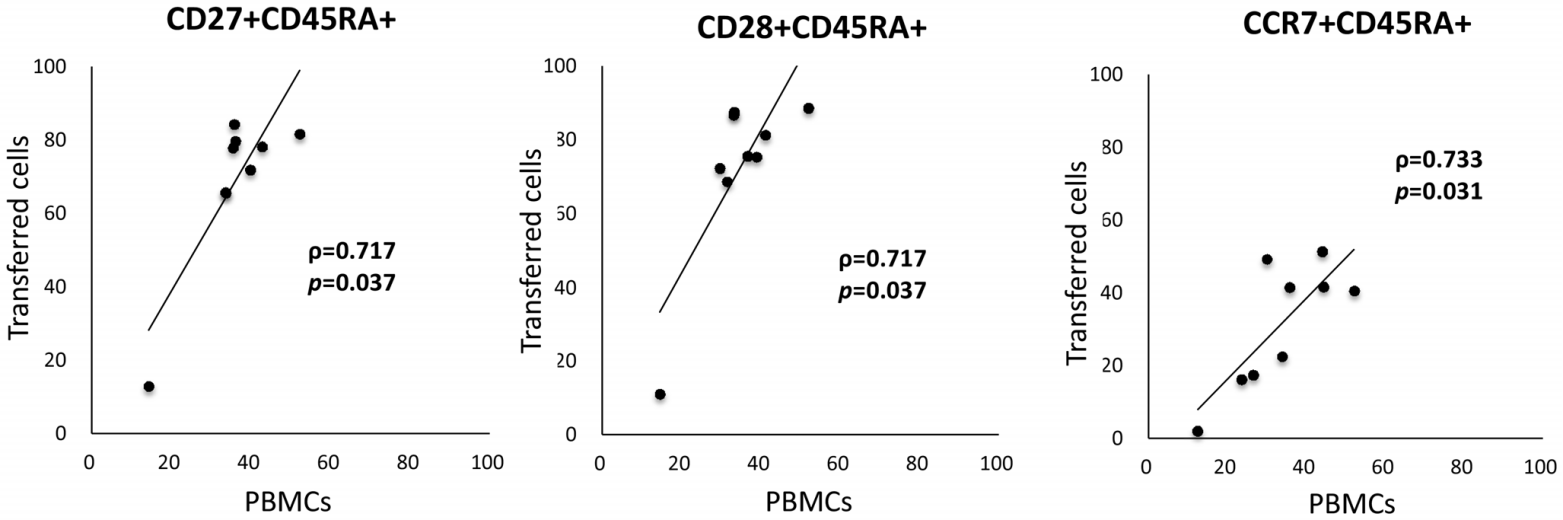
Correlation between the number of less-differentiated T-cell surface markers of transferred cells (after culture) and those of PBMCs (before culture). The comparison was done in terms of cell-surface markers (i.e. CD27+CD45RA+, CD28+CD45RA+, CCR7+CD45RA+).

Cells expanded a mean of 394.0-fold (range, 292.5-554.5-folds) during the 10-day culture period. The mean cultured cell viability was 97.4% (range 95.7-98.5%). Changes in cell-surface phenotype after culture are shown in Figure 2. Before stimulation, PBMCs contained approximately 45% CD4+ cells and 25% CD8+ cells. The ratio of CD8 cells significantly increased to almost 60% after culture, whereas CD4+ cell ratio showed a decrease. The ratio of CD27+CD45RA+ and CD28+CD45RA+ cells which was expressed in less-differentiated T cells, significantly increased to almost 60% after culture, whereas there was no significant change in the ratio of CCR7+CD45RA+ cells. Both CD3+HLA-DR+ and CD8+NKG2D+ cell population which are activation markers for lymphocytes, significantly increased after culture. On the other hand, CD3-CD56+ (NK cell) and CD3+CD56+ cell population (NKT cell) were insignificant after culture (mean of 0.73% and 2.16% respectively). The transferred cell phenotype of each case is shown in Table 2. The large majority of transferred cells were CD3 positive (98.1% as mean), and there were very few CD3-CD56+ cells (0.73% as mean). The ratios of CD4+, CD8+, and CD8+NKG2D+ cells in transferred cells were 36.2%, 60.3%, and 53.7%, respectively. As for less-differentiated T cell phenotype, CD27+CD45RA+, CD28+CD45RA+, and CCR7+CD45RA+ cells were 59.5%, 60.0%, and 22.4%, respectively. The proportion of these less-differentiated T cell phenotype was markedly different among patients, particularly, the proportion in patient 2 was considerably low (Table 2). Ratios of CD27+CD45RA+, CD28+CD45RA+, and CCR7+CD45RA+ transferred cells (after culture) strongly correlated with those of PBMCs (before culture) (Fig. 3).

**Table 2:** Characteristics of infused cells.

Patinet No.	CD3^+^	CD4^+^	CD8^+^	CD3^+^HLA-DR^+^	CD8^+^NKG2D^+^	CD3-CD56^+^	CD3^+^CD56^+^	CD27^+^CD45RA^+^	CD28^+^CD45RA^+^	CCR7^+^CD45RA^+^
1	99.45	28.41	70.09	81.87	63.27	0.38	1.99	77.83	75.57	41.67
2	96.52	41.37	53.51	80.10	42.52	2.33	6.32	12.91	11.03	2.03
3	98.35	44.16	52.14	87.18	45.02	1.15	1.19	35.37	33.64	9.87
4	99.41	26.83	68.72	91.74	59.93	0.39	1.65	68.25	54.41	28.33
5	99.68	41.21	53.48	93.18	50.06	0.36	1.62	56.06	63.96	16.67
6	99.50	44.85	48.92	85.80	45.19	0.41	2.65	57.03	60.33	16.47
7	99.30±0.47	27.59±2.53	70.875±1.62	91.16±0.06	62.97±3.17	0.55±0.33	1.37±0.37	65.66±0.06	70.45±2.59	16.84±0.87
8	99.74±0.06	21.37±3.56	77.27±3.58	86.22±7.26	68.98±4.86	0.08±0.04	1.00±0.19	85.25±1.36	88.46±2.47	25.44±4.16
9	95.88±4.82	50.21±8.12	47.50±8.26	82.04±2.48	44.92±8.52	0.96±0.69	1.66±1.26	77.22±4.96	81.74±6.64	44.56±5.91

Patient no. 1 to 6 needed a one-time culture and patients 7 to 9 underewent three lymphocyte cultures.

Values are expressed as mean±SD.

### Toxicity

The adverse events reported in this study are shown in Table 3. One patient, who was administered S-1, experienced grade 3 neutropenia however, this episode was considered to be related to S-1 and not to ACT. Grade two or lower level hematologic toxicities were observed mainly in patients administered with S-1, and these hematological toxicities were mostly transient. Non-hematological events including fatigue and anorexia were observed, but these were all mild. No ACT-related severe or unexpected toxicities were observed. Thus, no DLT was observed in this phase I study.

**Table 3:** Maximum toxicity per patient.

	Any Grade (with S-1)	Grade 3–4 (with S-1)
Hematological		
Neutropenia	3 (3)	1 (1)
Lymphopenia	2 (1)	0
Anemia	5 (5)	0
Thrombocytopenia	8 (6)	0
Increased aspartate aminotransferase	1 (1)	0
Increased alanine aminotransferase	1 (1)	0
Increased alkaline phosphatase	3 (3)	0
Increased total bilirubin	6 (5)	0
Non-hematological		
Fatigue	3 (3)	0
Anorexia	3 (3)	0
Nausea	1 (1)	0
Diarrhea	2 (2)	0
Mucositis	2 (2)	0

### Clinical Outcome

One patient (no.4) achieved complete response (CR). The CR patient suffered from lymph node recurrence after a rectal cancer operation and she was treated with mFOLFOX6 as first line chemotherapy. Subsequently, she was treated with Capecitabine as second line chemotherapy, but intrapelvic lymph node metastasis was detected by FDG-PET. The patient then received ACT therapy combined with S-1. Three months after 6 infusions of cultured T cells, the lesion completely disappeared. Patient no.8 experienced partial response (PR). He had refractory bile duct cancer, and was treated with GEM as first line chemotherapy followed by GEM/S-1 as 2^nd^ line chemotherapy. However, 17 months after starting chemotherapy, the size of the primary tumor increased and metastases in the cervical vertebra were detected. He then underwent ACT therapy combined with S-1. Two months after 6 infusions of ACT, the size of the primary tumor in his liver decreased by 35% and metastatic lesions in the cervical vertebra completely disappeared by FDG-PET.

Of the 9 patients, one (11.1%) exhibited CR, one (11.1%) had PR, 4 (44.4%) had stable disease (SD), and 3 (33.3%) had progressive disease (PD) (Table 4). The response rate was 22.2% (95% confidence interval (CI) 2.8 ? 60.0%) and the disease control rate (DCR; CR+PR+SD) was 66.7% (95% CI 29.9 – 92.5%).

**Table 4:** Tumor response.

No. of patients	Response				Response rate (%) (95% CI)
	CR	PR	SD	PD	
9	1	1	4	3	22.2 (2.8–60.0)

CR = complete response; PR = partial response; SD = stable disease; PD = progressive disease; 95% CI = 95% confidence interval.

### Immune Monitoring

**Figure 4:**
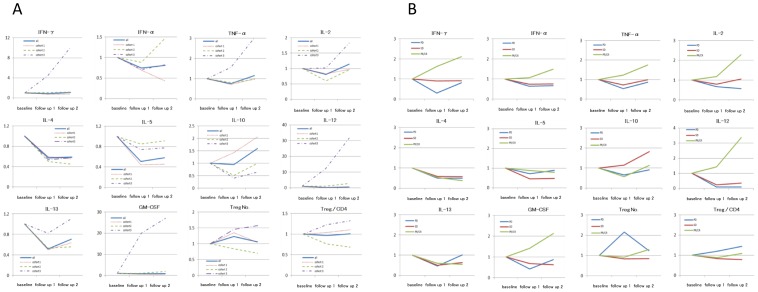
Longitudinal plots of whole blood cytokine levels plotted according to the deviation of cytokine levels from the baseline. Mean cytokine levels in subjects in each cohort (A) and levels for the various tumor responses (B) are shown.

To determine the immune responses in patients receiving novel ACT, whole blood cytokine assays and peripheral Treg analysis, which could be useful in immune monitoring[18]–[20], were done using venous blood obtained from patients. There was no marked change in whole blood cytokine levels (Fig. 4A) in patients after they were infused with cultured cells; however, there was a decrease in IL-4, IL-5 and IL-13 after the treatment. The levels of IFN-γ, TNF-α, IL-2, IL-12 and GM-CSF in cohort 3 increased after the treatment; in particular, the levels of IFN-γ, IL-12 and GM-CSF increased a multiple of 10 or more times. Next, we evaluated whole blood cytokine levels according to objective tumor response. The levels of IFN-γ, IL-2, IL-12 and GM-CSF in patients with PR/CR increased more than twice after the treatment, while those levels in patients with SD or PD decreased or did not change significantly post treatment (Fig. 4B).

As for Tregs, both the number and proportion of these cells in the peripheral blood showed no significant change after treatment when they were evaluated according to cohort or tumor response (Fig. 4A, B).

## Discussion

The results presented in this study indicate that FN-CH296-stimulated T cell therapy was very well tolerated in our patient sample. No significant ACT-related grade 3 or 4 adverse events occurred in any patient and the ACT produced tumor regression in 2 patients: CR in one patient with rectal cancer and PR in another with bile duct cancer. The response rate was 22.2% and the DCR was 66.7%. Despite the size of the sample, these results are promising.

The findings of previous mouse studies and clinical trials demonstrated that less-differentiated T cells may be the optimal population for ACT-based immunotherapies because of their in vivo persistence, high proliferative potential, receptiveness to homeostatic and costimulatory signals, their homing to secondary lymphoid tissues and their ability to secrete IL-2 [2]–[5]. Added to this, Yu et al. have reported that FN-CH296 acting with anti-CD3 induce T cell proliferation. These evidence suggests thats FN-CH296/anti-CD3 stimulation can be an efficient way to generate a large number of less-differentiated T cells suitable for ACT resulting in higher and longer persistence in vivo [13].

CD45RA, CD27, CD28, and CCR7 are known to be highly expressed in less-differentiated T cells. Based on human viral infection studies, a linear model of T cell differentiation has been proposed wherein CD27+CD28+CD45RA+ naïve cells progress through a CD27+CD28+CD45RA- early antigen-experienced phenotype and then proceeds to a CD27+CD28-CD45RA−/+ intermediate phenotype and finally to CD27-CD28-CD45RA+/− late antigen-experienced phenotype. This linear movement leads to an increase in cytotoxic potential and a reduction in cell proliferation [21]. CD27 and CD28 are costimulatory molecules that act in concert with T cell receptor (TCR) to support T cell expansion [22]. It has been suggested that CD28-expressing T cells secrete IL-2 and induce antiapoptotic molecules [23]. In recent times, the role of CD28 expression in ACT-based clinical trials has been getting closer attention and more detailed investigations are being done. Analysis of persisting and non-persisting TIL clones indicate preferential survival of the clonotypes expressing high levels of CD28, implicating a survival advantage for transferred T cells with CD28+ less-differentiated phenotype [4], [5]. CD27 can also augment TCR-induced T cell proliferation and is required for the generation and maintenance of memory T cells in vivo [24]. There is evidence that the frequency of T cells expressing CD27 increases gradually after ACT and may be associated with the long-term maintenance of a stable number of tumor-specific T cells in responding patients [25]. In this phase I clinical trial, the frequencies of both CD27+CD45RA+ and CD28+CD45RA+ cells increased significantly after culture and their population in transferred cells were about 60%. On the other hand, the frequency of CCR7+CD45RA+ cells, which were also expressed in less-differentiated T cells, did not change after culture for unknown reasons. The ratios of the less-differentiated phenotype T cells (i.e. CD27+CD45+, CD28+CD45RA+, and CCR7+CD45RA+ cells) in transferred cells differed greatly for each patient. A strong positive correlation was found between the ratios of CD27+CD45RA+ (ρ = 0.717, p = 0.037), CD28+CD45RA+ (ρ = 0.717, p = 0.037), CCR7+CD45RA+ (ρ = 0.733, p = 0.031) cells in transferred cells and those in PBMCs (before culture). It is necessary to improve upon or find new methods that can efficiently generate large numbers of less-differentiated T cells even in cases where there are few less-differentiated T cells. Consistent with previous reports [13], we found that stimulation with FN-CH296/anti-CD3 preferentially expanded CD8+ cells in this study. It is generally believed that the predominant tumoricidal effector mechanism is the cytotoxic killing effect of CD8^+^ T cells, however, despite the research done to date, the impact of the preferential proliferation of CD8 T cells on cancer immunotherapy still needs much further investigation.

Previous studies have demonstrated that IFN-γ plays an important role in cancer immunotherapy and IFN-γ expression of T cells is considered to be highly correlated with therapeutic success. We have also demonstrated that the assay of whole blood IFN-γ levels was an efficient method for evaluating clinical response to cancer immunotherapies [19] [20]. In this study, whole blood IFN-γ levels in cohort 3 increased up to 10 or more times after 6 infusions of cultured cells. Whole blood IFN-γ levels in PR or CR cases increased more than twice, whereas those in PD or SD cases saw no significant increase after the treatment. The finding in our prior study [20] that the increase in whole blood IFN-γ levels after ACT was independently related to overall survival in cancer patients emphasizes the relevance of the results gained in this study.

While no significant correlation between the number of infused less-differentiated T cells and whole blood IFN-γ levels was not confirmed probably due to small sample sizes, we surmise that less-differentiated T cells may contribute to the elevation of IFN-γ levels. Future studies are needed to explore the effect of the number of CD27+CD45RA+, CD28+CD45+ and CCR7+CD45RA+ cells on the whole blood IFN-γ levels.

In addition to IFN-γ other Th1 cytokine levels such as IL-2, IL-12 and TNF-α also increased in patients in cohort 3, and in patients with PR/CR, whereas Th2 cytokine levels such as IL-4, IL-5 and IL-13 decreased after the treatment. Besides, whole blood GM-CSF level increased in patients in cohort 3 and in patients who experienced PR/CR. It was previously reported that cell-mediated immunity is preferentially activated by Th1 cytokines, whereas Th2 cytokines suppresses cell-mediated immunity [26]. GM-CSF is a pleiotropic cytokine that stimulates dendritic cells (DCs) and promotes uptake of tumor antigens by DCs leading to T-cell cross-priming and activating the immune system against specific antigens [27], [28]. Consequently, based on the results from the immune-monitoring done in this present study, we are inclined to believe that the fibronectin CH296-stimulated T cell therapy may exert anti-tumor effects by activating cell-mediated immunity. Although we recently demonstrated that ACT has the potential to reduce the number of Tregs [29], in the analysis done in this study, the number of peripheral Tregs did not change significantly post treatment.

Responsiveness to immune-based therapies varies among tumor types. While melanoma and renal-cell cancer are classically considered to be immunogenic, most epithelial malignancies are not immunogenic and respond poorly to immunotherapy. In our sample, all nine patients had epithelial malignancies such as gastrointestinal cancers and lung cancer which are traditionally considered as refractory to immunotherapy. It should be noted that objective response was observed in patients with rectal cancer and bile duct cancer. It remains unclear if this novel ACT has therapeutic potential for multiple tumor types. Additional studies are needed to determine the breadth of activity of this novel ACT in human malignancies.

In this phase I clinical trial, we demonstrated the safety of FN-CH296-stimulated T cell therapy in patients with advanced cancer. The results were promising with a response rate of 22.2% and DCR of 66.7%. ACT technology is a platform with high potential that can be further improved. Modification of T cells with transgenes encoding T cell receptors (TCRs) or chimeric antigen receptors (CARs) allows tumor specificity to be conferred on functionally distinct T cell subsets [30], [31]. FN-CH296 stimulation could be efficient at generating large numbers of engineered T cells with less-differentiated phenotype. We surmise that T cell expansion using FN-CH296 could be applicable to various T cell based therapies such as engineered T cell therapy.

## Supporting Information

Protocol S1 **Trial protocol.**
(DOCX)

Flowchart S1 **CONSORT Flow Diagram.**
(DOC)

Checklist S1 **CONSORT checklist.**
(DOCX)
