# Supplementary material for: Phase I Clinical Trial of Fibronectin CH296-Stimulated T Cell Therapy in Patients with Advanced Cancer
Source: PLoS One. 2014 Jan 31;9(1):e83786. doi: 10.1371/journal.pone.0083786 (PMC3908868; doi:10.1371/journal.pone.0083786)
Supplement: Protocol S1 — Trial protocol. (DOCX) [file pone.0083786.s001.docx]

**Scientific title of the study**

Phase1 study of adoptive cell transfer therapy for advanced cancer patients, using stimulated T cells by CH-296.

**Condition**

unresectable digestive cancer, lung cancer

**Objectives**

To evaluate adverse events following adoptive CH-296-stimulated T cell transfer for advanced digestive cancer and lung cancer

**Assessment**

**Primary outcome**

Adverse events

**Secondary outcome**

Response rate

**Study design**

Single-arm, non-randomized, open, uncontrolled study

**Interventions**

Intravenous transfer of autologous T cells.
Dose-escalation study with three groups
Cohort 1; 1x10*9 cells 3 patients
Cohort 2; 3x10*9 cells 3 patients
Cohort 3; 9x10*9 cells 3 patients

**Eligibility**

**<inclusion criteria>**

1) Histologically confirmed digestive cancer or lung cancer.
2) Patients informed diagnosis of their disease
3) Patients must have recurrent disease after standard treatment, and in the situation where curative treatment opions are not available.
4) Patients must have residual disease after standard treatment, and in the situation where curative treatment options are not available.
5) Without planning to recieve chemotherapy, radiation therapy or BRM. Oral 5-FU drugs are permitted.
6) Aged from 20 to 80 years
7) Grade 0 to 2 in performance status(ECOG).
8) Expected to be alive at least three months after informed consent.
9) Lasting at least four weeks since the previous chemotherapy or radiotherapy, or two weeks since the previous administration of 5-FU. But oral 5-FU drugs are permitted.
10) Sufficient functions of major organ.
11) Having written informed consent.

**<exclusion criteria>**

1) Presence of uncontrolled infection
2) Hypersensitivity or autoimmune disease requiring treatments
3) Presence of severe comlications(malignant hypertension, congestive heart failure, severe coronary disease, history of myocardial infarction less than six months prior, pulmonary fibrosis or active interstitial pneumonitis)
4) Inappropriate patients for this study due to severe complications.
5) Active other malignancy, except lesions of "carcinoma in situ" or intramucosal location which are curatively resectable.
6) Medical history of severe hypersensitivity.
7) Severe mental impairment
8) Pregnant or lactating women
9) Inappropriate patients for study judged by the physicians

**Target sample size**

9

**実　　施　　計　　画**

| **1 課題名**  　　ナイーブT細胞リッチTリンパ球移入療法の再発・進行癌に対する第I相臨床試験  **2 実施計画の意義、目的及び方法**   1. **目的**   レトロネクチン等を用いた培養Tリンパ球を進行・再発消化器癌、肺癌に反復投与し、その安全性を評価することを主目的とする。  ・主要エンドポイント：培養Tリンパ球の反復輸注の安全性について、有害事象の種類、程度、頻度を評価する。  ・副次的エンドポイント：腫瘍縮小効果を評価する。   1. **背景と問題点**   高度進行悪性腫瘍に対する現状の治療には限界があり、治療効果の改善のため免疫治療が期待されている。しかし、これまでに試みられてきたがんに対する免疫療法の多くは、個別には奏功する例が見られるのも事実であるが、全体としてみればその効果は不確実なものであった。  　現在行われている養子免疫療法に用いられるCD3-LAK細胞は、体内へ輸注後の寿命が短いことが、動物モデルにおいて確認されており、そのことがCD3-LAK療法の治療効果を限定的なものとしている１つの要因と考えられている。動物モデルにおいては、ナイーブT細胞のような未分化なリンパ球の方が、in vivoにおいて高い抗腫瘍活性を示すことが確認されており、ナイーブT細胞は養子免疫療法の移入細胞として、より適した細胞と考えられている。  　本研究では、養子免疫療法に用いられるリンパ球としてより適していると考えられるナイーブT細胞を多く含む、レトロネクチンを用いて培養したTリンパ球の安全性と有効性を評価し、臨床的応用を目指す。   1. **試験細胞情報**   培養Tリンパ球は、末梢血より分離したTリンパ球を抗CD3抗体とレトロネクチン(CH-296)により初期刺激を行い、IL-2を添加して拡大培養したものである。刺激時にレトロネクチンを加えることで、拡大培養率が高くなり、得られてくるTリンパ球集団はナイーブT細胞の表現型として知られるCCR7陽性CD45RA陽性細胞を多く含むことが明らかとなっている。拡大培養後のCCR7陽性CD45RA陽性細胞は、サイトカイン産生能、ケモカイン反応性、抗原特異的CTLへの分化能などの試験においてナイーブT細胞と同様の機能を持っていることが確認されている。  　　3.1.試験細胞の剤形および含有量  試験細胞の含有量は、  a) 1×10^9^ cells  b) 3×10^9^ cells  c) 9×10^9^ cells  の3通りである。試験細胞は乳白色である。  　　3.2.試験細胞の包装形態  フローズバッグに、   1. 1×10^9^ cells投与用には50ml(2×10^7^ cells/ml) 2. 3×10^9^ cells投与用には50ml(6×10^7^ cells/ml) 3. 9×10^9^ cells投与用には90ml(1×10^8^ cells/ml)   とする。   1. **適格基準**   4.1　選択基準  a) 組織診において消化器癌（食道癌、胃癌、結腸癌、直腸癌、膵癌、胆道癌、肝細胞癌）または肺癌であることが確認されている症例  b) 上記病名を告知されている症例  c) 標準治療後の再発例で、手術・放射線治療による根治性のある治療の対象とならない患者  d) 標準治療後の腫瘍残存例で、手術・放射線治療による根治性のある治療の対象とならない患者  e) 化学療法、放射線療法、BRM(Biological Response Modifiers)療法を施行されていない症例。ただし、5-FU系経口抗がん剤の服用は登録可能とする。  f) 登録日時点の年齢が20歳以上、80歳未満の症例  g) Performance Status(ECOG版)が0-2の症例  h) 3ヶ月以上の生存が期待できる症例  i) 前治療（化学療法、放射線療法）のある患者においては、wash-out期間は原則として4週間以上(5-FU系薬剤は2週間以上)とし、前治療の効果や有害事象による影響を持ち越していないと判断される症例。ただし、5-FU系経口抗がん剤の服用の症例では、wash-out期間を設けない。  j) 主要臓器機能が保持されている症例  末梢血所見：白血球数　3000/μl以上　12000/μl以下  好中球数　1500/μl以上  血小板　100000/μl以上  ヘモグロビン　9.0g/dl以上  肝機能：血清AST・ALT　100IU/l以下  総ビリルビン値　2.0mg/dl以下  腎機能：血清クレアチニン値　1.5mg/dl以下  心電図：重篤な異常を認めない  k) 本試験参加について文書による同意が本人より得られた症例  4.2.除外基準  a) コントロール不良な感染症を有する症例  b) 治療を必要とするアレルギーまたは自己免疫疾患を有する症例  c) 重篤な合併症（悪性高血圧、重症のうっ血性心不全、重症の冠不全、6ヶ月以内の心筋梗塞、重症の肺線維症、活動性の間質性肺炎等）を有する症例  d) 合併症のために本試験への参加がふさわしくないと考えられる症例もしくは重大な医学的事象のために安全性が損なわれる可能性のある症例。ただし、NCI-CTC ver 3.0 日本語訳JCOG版に規定するgrade3以上を目安とする。  e) 腸管麻痺、腸閉塞を有する症例  f) 多量の癌性体腔液（胸水、腹水、心嚢水）を有する症例。  g) 症状を有する脳転移のある症例  h) 活動性の重複癌を有する症例（局所治療により治癒と判断されるcarcinoma in situまたは粘膜内癌相当の病変は活動性の重複癌に含めない）  i) 重度の薬剤過敏症の既往歴を有する症例  j) 重度の精神障害を有する症例  k) 妊娠中、授乳中の症例  l) その他、研究責任医師または研究分担医師が不適当と判断した症例。   1. **被験者の登録手順**   研究責任医師または研究分担医師は、「登録用紙」に必要事項を記入の上、研究事務局に提出する。研究事務局は、「登録用紙」の記載に基づいて適格性を確認する。適格と判定された場合、研究事務局は登録番号を割り当てのうえ登録を行う。  適格と判定された場合、研究責任医師または研究分担医師は培養Tリンパ球の調製を開始する。   1. **予定登録症例数と実施期間**   予定登録症例数：9例  実施期間：承認日　～　平成２２年　３月 ３１日   1. **治療計画と用量**   7.1. プロトコル治療  7.1.1. 試験細胞の調製及び投与  　採血により得られた血液より末梢血単核球（PBMC）を分離し、試験細胞の調製を行う。  　採血及び試験細胞の調製は１回で必要量確保できない場合、必要に応じて随時行う。  a) 採血  京都府立医科大学消化器内科において、静脈血を50～100mL以上採取する。  予め、研究事務局は、研究担当医師、細胞調製室と採血に関するコーディネートを行い、培養Tリンパ球調製依頼書にて、細胞調製を依頼する。研究事務局は採血情報を、細胞調製用採血確認書、検体搬入依頼書を用いて細胞調製室、研究担当者へ連絡する。所定の日時に採取された血液は検体情報と共に、研究担当医師の指示を受けたもの（細胞調製室のスタッフ）が適切な容器に入れ、京都府立医科大学から医聖会百万遍クリニック（京都市左京区田中門前町103-5 京都パストゥール研究所ビル１Ｆ）に輸送する。百万遍クリニック内の細胞調製室へ搬入する。  b) 細胞調製室  　　試験細胞の調製にかかわる全ての操作は、GMP（薬剤作製の基準）に準じて、厳密に管理された細胞調製室（CPR）で行う。　または可能な工程については閉鎖系で細胞を取り扱うことにより、他の患者由来の細胞や外来微生物の混入を防止する。  c) 試験細胞の培養  試験細胞(培養T細胞)の調製工程の概略は以下に示すとおりである。  第0日：被験者血液よりPMBCを分離・洗浄後、培養用培地にPBMCを懸濁し、生細胞数・生存率を測定する。予め抗CD3抗体及びレトロネクチン(CH-296)を固定化した培養用バッグに移し、IL-2を終濃度200U/mLとなるように添加して、培養を開始する。  第4日：何も固定化していない新しい培養用バッグに細胞懸濁液を移し、新しい培地及びIL-2を終濃度200U/mLとなるように添加して、培養を継続する。  第7日：新しい培地及びIL-2を終濃度200U/mLとなるように添加して、培養を継続する。  第10日：細胞を洗浄・濃縮・回収する。凍結保存用溶液に濁液した後、フローズバッグに移し、投与するまで凍結保存する。  d) 試験細胞の保存  　　 試験細胞は、百万遍クリニック内の細胞調製室で凍結保存された後、細胞情報と共に京都府立医科大学消化器内科へ実施担当者の指示を受けたもの（細胞調製室のスタッフ）が、閉鎖系のバッグで凍結し、ドライアイス入りの容器に入れ、百万遍クリニックから京都府立医科大学に輸送し、医師（実施担当者）に手渡す。搬入後の試験細胞は、京都府立医科大学内で管理された冷凍庫で使用時まで-80℃で凍結保存される。  e) 試験細胞の投与  　　品質試験で全ての試験項目に合格した場合のみ、各投与グループにて設定された細胞数の試験細胞を静脈内投与する。投与前に凍結保存された試験細胞を37 ℃恒温槽で急速解凍し、適宜生理食塩水を添加してから、被験者へ投与する。投与直後より、問診とバイタルサインを取りながら、十分な観察を行ない、有害事象が発現した場合には、「4　予測される結果及び危険性」に従い、適切な処置を施す。  f) 試験細胞の品質管理  試験細胞を調製する百万遍クリニック内の細胞調製室は不純物（雑菌等）混入を最小限とする環境に管理されている。細胞調製の際に用いられる培地由来の成分、試薬、抗体等に関しては、被験者に投与される前に十分に洗浄され、実際に被験者に投与される量は極めて少ない。試験細胞の品質は、培養終了後以下の品質試験を行うことにより担保する。  試験細胞品質試験項目及び判定基準  ①細胞濃度試験：各投与グループ設定値の80%－120%の範囲内  ②細胞生存率試験：80%以上  ③免疫表現型試験(CD3陽性細胞率) ：90％以上  ④エンドトキシン試験：1.56pg/mL以下  ⑤無菌試験：適合  ⑥その他の免疫表現型試験（試験細胞の性質確認の補助的試験としてフローサイトメトリーにより細胞表面マーカーの確認を行う。）  　これらの試験は投与されるまでの凍結保存中に行われる。品質試験で全ての試験項目に合格した場合だけ、試験細胞を投与することとする。  7.1.2. 治療スケジュール  試験細胞の投与は2週間隔で計2回投与する。2回目投与後4週目に試験を終了し安全性を評価する。  7.1.3. 症例数  9例(各グループ：3例)  グループA：試験細胞、1×10^9^ cellsを投与  グループB：試験細胞、3×10^9^ cellsを投与  グループ C：試験細胞、9×10^9^ cellsを投与  なお、1症例中の用量変更は行わない。  　　7.1.4. 予定期間  実施期間　　承認日　～　平成２２年　３月 ３１日  　　7.1.5. 臨床研究の中止  以下の場合は研究を中止する。研究を中止した際は、投与中止報告書にて研究事務局へ報告し、症例報告書に記録する。  a) NCI-CTC ver 3.0グレードにて非血液毒性のグレード3以上の有害事象発生時には中止する。ただし、グレード3以上の発熱、食思不振、下痢、口内炎、悪心、嘔吐、発熱性好中球減少については、7日以内に回復した場合は中止しない。その他のグレード3以上の有害事象はコースの終了時に、効果安全性評価委員会にて試験で併用される化学療法剤に起因することが“確からしい”と判断されれば、中止せずに次コースを行う。  b) 被験者が同意を撤回した場合中止とする。  c) プロトコル開始後、不適格症例であると判明した場合中止する  d) その他、研究責任医師または研究分担医師が治療の継続が不適切であると判断した場合中止する  　　7.2. 後治療  2回目の試験細胞投与後4週目に試験を終了し、安全性評価を行う。投与の継続に安全上問題がないと判断され、被験者が希望した場合には、その被験者に対して同一プロトコルで試験細胞をさらに４回繰り返し投与を行える。ただし、一症例中の試験細胞数の変更は行わない。   1. **有害事象の評価・報告**   8.1. 有害事象の定義  　有害事象とは、本治療が実施された被験者に生じるあらゆる好ましくないあるいは意図しない症状、徴候(臨床検査の異常も含む)または病気のことであり、当該治療との因果関係の有無は問わない。  　重篤な有害事象とは、以下のものをいう。  a) 死亡にいたるもの  b) 死亡につながるおそれのあるもの  c) 治療のために入院または入院期間の延長が必要なもの  d) 障害をきたすもの（永続的または顕著な障害もしくは機能不全に陥るもの）  e) 障害につながるおそれのあるもの  f) 上記1)から5)に準じて重篤であるもの  g) 後世代における先天性の疾病または異常をきたすもの  8.2. 有害事象の評価  　発現した有害事象のグレードは、NCI-CTC ver 3.0 日本語訳JCOG版に従い、判定を行う。  8.3. 有害事象の報告  報告義務のある有害事象は、11.1.有害事象の定義で規定した「重篤な有害事象」のうち、プロトコル治療中またはプロトコル治療終了後（30日以内）に発生したものとする。   1. **投与量の変更(dose escalation)**   目標症例数は以下の通り各投与グループ3例ずつの計9例とするが、有害事象が発現した場合には、必要に応じてその投与グループの症例数を増加し、安全性の評価を強化する。  1回当たりの培養Tリンパ球輸注量   1. 第一段階投与グループ 1×10^9^ cells 3例 2. 第二段階投与グループ 3×10^9^ cells 3例 3. 第三段階投与グループ 9×10^9^ cells 3例   培養Tリンパ球数の設定  ①3例のうち1例も「本臨床研究の中止基準に定める中止基準を満たす本治療との因果関係を否定できないグレード3以上の有害事象」が発現しなかった場合、次の投与グループに3例が登録される。  ②3例のうち1例に「本臨床研究の中止基準に定める中止基準を満たす本治療との因果関係を否定できないグレード3以上の有害事象」が発現した場合、同じ投与グループに3例が登録される。  ③3例のうち2例または3例に「本臨床研究の中止基準に定める中止基準を満たす本治療との因果関係を否定できないグレード3以上の有害事象」が発現した場合、投与量の増加はそこで中止される。  ④上記2)に従った後、6例のうち1例に「本臨床研究の中止基準に定める中止基準を満たす本治療との因果関係を否定できないグレード3以上の有害事象」が発現した場合、投与量の増加は続けられる。  ⑤上記2)に従った後、6例のうち2例に「本臨床研究の中止基準に定める中止基準を満たす本治療との因果関係を否定できないグレード3以上の有害事象」が発現した場合、投与量の増加はそこで中止される。  また、試験細胞である培養Tリンパ球の培養中の増殖率は、培養ごとに予測することができないため、症例の取り扱いを以下の通りとする。  ①第一段階投与グループで定められた培養Tリンパ球輸注量( 1×10^9^cells)を本臨床研究における最小輸注量とする。最小輸注量に満たない場合には、培養Tリンパ球を投与せず、本臨床研究における脱落例とする。  ②第二段階投与グループで定められた培養Tリンパ球輸注量( 3×10^9^cells)が得られなかった場合には、その被験者に輸注可能な全ての培養Tリンパ球を投与する。投与した培養Tリンパ球輸注量が2.4×10^9^cells以上の場合、第二段階投与グループの症例数として数えるが、それ未満の場合は投与量増加の評価例としない。  ③第三段階投与グループで定められた培養Tリンパ球輸注量( 9×10^9^cells)が得られなかった場合には、その被験者に輸注可能な全ての培養Tリンパ球を投与する。投与した培養Tリンパ球輸注量が7.2×10^9^cells以上の場合、第三段階投与グループの症例数として数えるが、それ未満の場合は投与量増加の評価例としない。  ④投与量増加の評価対象としない症例数は、第二段階、第三段階投与グループにおいて最大3例までとし、その症例をもって当該投与グループへの登録を終了する。その後、研究責任者が全てのデータを確認し、効果安全性評価委員会と協議して次の方針を決定する。  投与量の移行の判断は、効果安全性判定委員会において行う。   1. **観察・検査・報告項目とスケジュール**   10.1. 治療期間・追跡期間の定義  a) 各患者の治療期間は、初回試験細胞投与開始日から2回目投与の4週後までとする。  b) 各患者の追跡期間は治療期間終了後3ヵ月とする。  10.2. 患者特性の調査  患者について以下の内容を治療開始前に調査・確認する。  ①性別  ②年齢（生年月日）  ③体重  ④身長  ⑤病理診断名  ⑥確定診断の方法  ⑦前治療の有無、内容、最終治療日または無治療期間  ⑧TNM分類  ⑨標的病変及び非標的病変、がん性体腔液の有無  ⑩合併症の有無と疾患名  ⑪薬剤アレルギーの有無と薬剤名  10.3. Stagingの方法  Full stagingは症例登録前に実施し、登録前4週間以内のデータを用いるものとする。）  10.4. 自他覚症状  主に下記の毒性について自他覚症状を調査する。  PS、悪心、嘔吐、全身倦怠感、下痢、便秘、発熱（非感染性）、感染、発熱性好中球減少、呼吸困難、発疹、静脈炎、口腔（粘膜障害）、その他のgrade3以上の毒性  10.5. 登録前評価項目  　a) 患者特性( 10.2. 参照）  　b) Full staging(10.3.　参照) （登録前4週以内）  　c) 自他覚症状(10.4. 参照)、 （登録前1週以内）  d) 臨床検査  血液、生化学検査 （登録前2週間以内）  ①血液検査（WBC、WBC分画、Hb、Plt、RBC）  ②生化学検査 （TP、Alb、ALP、LDH、ALT、AST、BUN、Cre、T-Bil、Na、K、Cl、Ca、CRP）  ③ 腫瘍マーカー検査  当該被験者においてそれまでに上昇が確認されている腫瘍マーカー  ④免疫モニタリング(末梢血リンパ球のサイトカイン産生能＜IFN-α, IFN-γ,IL-4, IL-10, IL-12, TNFα＞、Treg)  ⑤心電図  10.6. 治療期間中の評価項目  a) 効果判定のための検査  CT （試験細胞2回目投与4週後）  b) 自他覚症状  自他覚症状 （2週毎）  体重 （2週毎）  c) 臨床検査  血液検査（WBC、WBC分画、Hb、Plt、RBC）　　　　（2週毎）  生化学検査（TP、Alb、ALP、LDH、ALT、AST、BUN、Cre、T-Bil、Na、K、Cl、Ca、CRP）　　　　　　　　　　　　　　　　　　　　　(2週毎)  d) 腫瘍マーカー検査　　　　（試験細胞2回目投与4週後）  　　 e) 免疫モニタリング　(末梢血リンパ球のサイトカイン産生能＜IFN-α, IFN-γ,IL-4, IL-10, IL-12, TNFα＞、Treg)　　（試験細胞2回目投与4週後）  10.7. 追跡期間中の評価項目  a) 画像検査  CT （4週毎）  b) その他の検査  自他覚症状のチェック、体重の測定、臨床検査等は必要に応じて実施する。  10.8. 再発が疑われた場合  随時必要な検査を行う。   1. **エンドポイントの定義**   11.1. 主要エンドポイント  　安全性の評価  a) 有害事象  有害事象とは、本治療が実施された被験者に生じるあらゆる好ましくないあるいは意図しない症状、徴候(臨床検査の異常も含む)または病気のことであり、当該治療との因果関係の有無は問わない。  重篤な有害事象とは、以下のものをいう。  ①死亡にいたるもの  ②死亡につながるおそれのあるもの  ③治療のために入院または入院期間の延長が必要なもの  ④障害をきたすもの（永続的または顕著な障害もしくは機能不全に陥るもの）  ⑤障害につながるおそれのあるもの  ⑥上記①から⑤に準じて重篤であるもの  ⑦後世代における先天性の疾病または異常をきたすもの  研究責任医師または研究分担医師は、本研究期間中に発現した有害事象について、その内容、発現日、程度、本研究の処置（継続・中止の別等）、その他の処置、転帰、転帰日、本研究との因果関係（明らかに関連あり、多分関連あり、関連あるかもしれない、関連なし）を調査する。なお、本臨床研究との因果関係を否定できない有害事象については、原則として消失または軽快まで追跡調査を行う。  b) 臨床検査値  　研究責任医師または研究分担医師は、臨床検査値の正常・異常について判定する。  　また、研究責任医師または研究分担医師は、臨床検査値の異常変動の有無について判定する。異常変動「有」とは、正常値→異常値、もしくは異常値→正常値の増強がみられた場合、その臨床的意義を考慮して判定する。なお、異常変動の有無の判定について、正常→異常値もしくは異常値の増強がみられ、かつ異常変動「無」と判断した場合には、その理由について、臨床経過をふまえて考察を行う。  　　11.2. 副次エンドポイント  a) 腫瘍縮小効果の判定  本研究では、RECIST効果判定基準の測定可能病変、測定不能病変の定義に従い、試験細胞投与前4週以内に画像検査を行い、標的病変、非標的病変を選択する。腫瘍縮小効果の判定は「RECIST効果判定基準」に準じて行い、2回目試験細胞投与後4週目に画像検査を施行し判定する。   1. **統計学的検討**   12.1. データの集積  登録症例のデータは、本研究の症例報告書を用いて登録事務局に集積する。  12.2. データの解析  データ入力、解析の実施は、研究事務局で行う。   1. **症例報告書の記入と報告**   13.1. 様式と提出期限  試験終了・中止後、研究責任医師または研究分担医師は、速やかに症例報告書作成し、研究事務局へ提出する。  　　13.2. 症例報告書の作成および提出  研究責任医師または研究分担医師は、提出時期にあわせて対応する症例報告書に必要な内容を記入する。直接記入したものを原本とし、その原本を速やかに研究事務局へ提出すること。ただし、研究責任医師または研究分担医師は、原本を事務局に提出する前に、そのコピーを作成し、研究終了まで保管すること。   1. **倫理的配慮**   14.1. 遵守すべき諸規則  本研究は、「ヘルシンキ宣言」ならびに「臨床研究に関する倫理指針」（厚生労働省告示第四百五十九号、平成16年12月28日）に則り実施される。  　 14.2. 倫理委員会  本研究は、実施に先立ち、倫理審査委員会において、臨床研究実施計画書、被験者の同意を得るのに使用される方法などの資料において審査を受け、承認を得た後に実施する。  　　14.3. インフォームド・コンセント  研究責任医師または研究分担医師は、患者に臨床研究について十分に説明しなければならない。患者が熟読し保管できるように、臨床研究の詳細を示した説明文書を患者に渡す。患者は情報を十分に考慮できる時間を与えられ、疑問点を確認した後で、同意文書に署名および日付を記入し、十分な説明を受けた上で完全に同意したことを示す。文書による同意が必須である。  　　14.4. 被験者の機密保持（第３者提供の制限）  被験者の登録および症例報告書における被験者の特定は被験者識別コード等で行うとともに、原資料の直接閲覧・取扱い等においては被験者のプライバシー保護に十分配慮する。また、研究責任者はあらかじめ被験者の同意を得ずに個人情報を第３者に提供してはならない。本臨床研究では、タカラバイオ株式会社が外部協力者として「培養Ｔリンパ球調製技術の提供と助言」に限定し、間接的に関与する。したがって、タカラバイオ株式会社の担当者が研究協力のために一部データを閲覧する予定であるが、治験と同様に被験者識別コードから被験者を特定する情報については、研究責任者が厳重に管理するものとする。また、事前にその旨を被験者に通知し、文書にて同意を取得する。  　14.5. 補償  本研究において有害事象を認めた場合には、速やかに適切な診断と処置を医療健康保険の範囲内で行う。しかしながら、本研究に起因した健康被害に対する補償金は支払われない。  　　14.6. 被験者の研究参加に伴う負担の軽減  本研究への参加に対する対価は支払われないが、本研究で用いるTリンパ球の調製に関連する費用は、京都府立医科大学消化器内科で負担され、被験者が負担する必要はない。   1. **プロトコルの変更**   プロトコルの変更は、研究責任医師より提出され、効果安全評価委員会での承認を得ることにより可能とする。変更プロトコルの研究は倫理委員会の承認を得ることとする。   1. **研究の中止と終了**   16.1. 個々の被験者に対する中止規定または中止基準  以下の場合、研究責任医師または研究分担医師は、直ちに試験細胞の投与を中止し、必要な場合、適切な処置を実施する。さらに、中止日、中止理由およびその詳細、中止に至った経緯や実施した処置等を症例報告書の所定の頁に記入する。  ①同意が撤回された場合  ②被験者の選択基準を満たしていないことが判明した場合  ③被験者の除外基準に抵触することが判明した場合  ④試験細胞との因果関係の有無にかかわらず有害事象発現のため、研究の継続が困難と研究責任医師または研究分担医師が判断した場合  ⑤来院予定日に来院せず、研究の継続ができなくなった場合  ⑥その他、研究の継続が適当でないと研究責任医師または研究分担医師が判断した場合  16.2. 研究施設での中止または中断  研究責任医師または研究分担医師が、研究を中止または中断した場合には、京都府立医科大学長に速やかに文書で通知し、詳細を説明する。  　　16.3. 研究の終了  研究責任医師は研究終了後、京都府立医科大学長に研究が終了した旨を文書で通知し、研究結果の概要を文書で報告する。   1. **研究実施計画書の遵守**   研究責任医師または研究分担医師は、患者の安全と人権を損なわない限りにおいて本研究実施計画書を遵守しなければならない。   1. **記録の保存および成績公表の方法**   記録の保存は、京都府立医科大学長が指名した保管責任者である京都府立医科大学大学院医学研究科消化器内科学准教授の古倉　聡が行う。保管責任者は適切な状態の下で、本研究終了後少なくとも5年間保存するものとする。  　成績の公表は、被験者の同意のもと、研究者全員の合意を得て行う。公表の際には、被験者のプライバシーに十分配慮し、個人情報が特定できないよう必要な措置を講じる。   1. **研究者**   19.1. 研究責任医師  吉川　敏一  京都府立医科大学大学院医学研究科　消化器内科学　教授  〒602-8566 京都市上京区河原町通広小路上る梶井町465  電話: 075-251-5519 FAX: 075-251-0710  19.2. 研究分担医師  古倉　聡 京都府立医科大学大学院医学研究科　消化器内科学　准教授  石川　剛 京都府立医科大学大学院医学研究科　消化器内科学　研修員  坂元直行　　　京都府立医科大学大学院医学研究科　消化器内科学　研修員  　　19.3. 外部協力者  榎　竜嗣 タカラバイオ株式会社バイオ研究所細胞医療部門 主任研究員  （培養Tリンパ球調製技術の提供と助言）  　　19.4. 研究事務局  京都府立医科大学医学部　研究事務局  担当事務員  連絡先  〒602-8566 京都市上京区河原町通広小路上る梶井町465  電話: 075-251-5519 FAX: 075-251-0710  緊急連絡先・時間外連絡先  京都府立医科大学大学院医学研究科　消化器内科学  古倉　聡  携帯電話：090-5245-0868 or 070-6507-7614  19.5. 効果安全評価委員会  効果安全評価委員会は、以下の委員から構成され、研究責任医師の諮問に応じ、  研究期間中に得られた安全性情報に基づき、研究の継続、変更または中止を研究責  任医師に答申する。  委員長  　京都府立医科大学医学研究科　血液・腫瘍内科学 教授  谷脇　雅史  　　　 委員  　京都府立医科大学医学研究科　内分泌・代謝内科学　教授  中村　直登  委員  　社会保険京都病院　消化器科　部長  　安藤　貴志  　　 19.6. 個人情報管理責任者  　京都府立医科大学大学院医学研究科　消化器内科学　准教授  　古倉　聡  　〒602-8566 京都市上京区河原町通広小路上る梶井町465  電話: 075-251-5519 FAX: 075-251-0710   1. **研究成果の発表**   研究結果の学会、誌上発表は、研究責任者、研究担当医師の合意の下で、筆頭者、共著者を決定する。研究プロトコル作成者、症例担当者、解析担当者で研究結果への貢献度で判断することとする。  　なお、研究結果の公表に関しては、被験者より、研究参加時に同意を得ることとする。  **3 実施期間**  　承認日　～　平成２２年　３月 ３１日  **4 予測される結果及び危険性**  　本研究でTリンパ球培養のために使用されるヒト組換えタンパク質レトロネクチンは、GMPグレードで製造されたものを使用する。レトロネクチンは、体外でレトロウイルスベクターを用いて遺伝子導入する際にも使用されている。ヒトを対象とした国内外の体外遺伝子治療の臨床試験にもレトロネクチンが使用されており、治療実績は200名を超えている。またレトロネクチンをTリンパ球培養に使用した臨床研究が三重大学医学部付属病院、中国の天津医科大学で実施中である。また、これまで行ってきた活性化リンパ球療法（CD3-LAK）の経験から、本研究においても自己の培養Ｔリンパ球を用いるため、安全性は高いと考えるが、予期される有害事象とその対処については以下のとおりである。  予期される有害事象   1. 発熱、発疹、アレルギー類似反応、infusion reaction等   　培養Tリンパ球の調製の時に使用する培地には、抗生物資（硫酸ストレプトマイシン）が含まれる。細胞調製の最終段階で除去する操作を行うが、一部残った抗生物質によりアレルギーが引き起こされる可能性がある。試験細胞を調製後、投与されるまで一旦凍結保存される。解凍したリンパ球を投与した際に、解凍に伴い一部崩壊した細胞内のサイトカイン、凍害予防剤等による発熱、悪寒、皮疹、関節痛、嘔気等をきたす可能性がある。対処法は、経過観察あるいは解熱鎮痛薬や抗ヒスタミン剤等の適切な薬剤を投与する。また、グレード3以上の場合には副腎皮質ステロイド剤の投与を行う。   1. 肺障害   　本研究は自己血液細胞輸注を行うため、輸血副作用として知られる輸血関連急性肺障害(TRALI: Transfusion-Related Acute Lung Injuruy)類似病態発症の可能性は考えにくいが、試験細胞投与後の肺障害に留意すべきと考えられる。対処法として、発症時には副腎皮質ステロイド剤の大量投与等、適切な処置を行う。   1. アルブミン製剤について   　本研究では、培養Tリンパ球を良い状態で投与するために、アルブミン製剤を含んだ保存用液に混合して使用する。アルブミン製剤は、特定生物由来製品に該当する血漿分画製剤のひとつで、その使用時には患者または家族から同意を得ることに関しては、薬事法68条の7に定められている。アルブミン製剤は、製造の過程で種々のウイルスの除去等を実施して品質管理に努め、その結果、輸血などと比較しても肝炎ウイルスなどの感染の危険性は減少している。しかし、人の血漿から有効成分を精製して製造されるため、未知の感染症を引き起こす可能性はわずかながら存在している。また、まれに、発熱や蕁麻疹などの過敏症を起こす可能性もある。本研究ではアルブミン製剤の使用について被験者に同意説明書にて説明を行い、同意を得て行われる。  **5 個人情報の保護の方法**  登録症例の同定は、登録時に発行される登録番号、症例イニシャル、生年月日、カルテ番号を用いて行い、これらの情報は、研究分担者の消化器内科准教授の古倉　聡が責任を持って管理する。情報管理にはパソコンを使用するが、このパソコンはネットワークから隔絶された状態で使用および管理される。  本研究は個人情報保護のために最大限の努力を行い、施設、研究事務局間での症例データのやりとりは、紙、電子媒体のいかんにかかわらず、症例登録を除き、直接手渡しすることを原則とする。  **6 研究対象者、試験提供者を選ぶ方針**  　手術不能・再発消化器癌（食道癌、胃癌、結腸癌、直腸癌、膵癌、胆道癌、肝細胞癌）および肺癌の患者で、根治的な治療法がなく、Performance Status(ECOG版)が0-2の症例であり、2-4)の症例適格基準を満たす患者。  **7 研究対象者の内容(年齢、性別、人数)、試料の内容(種類、量)**  2-4) 症例適格規準を満たす術不能・再発消化器癌（食道癌、胃癌、結腸癌、直腸癌、膵癌、胆道癌、肝細胞癌）および肺癌の患者、したがって20歳以上、80歳未満で性別は不問。  登録人数は、2-6) 予定登録症例数に示すとおり9名。  **8 共同研究機関の名称**  医聖会百万遍クリニック  （京都市左京区田中門前町103-5 京都パストゥール研究所ビル１Ｆ）  **9 実施責任者等**  　実施責任者：消化器内科　吉川敏一  　実施担当者：消化器内科　古倉　聡  **10 インフォームド·コンセントのための手続および方法**  1)　同意説明およびその他の説明文書  　研究責任者または研究分担医師は、被験者から研究への参加の同意を得るために用いる同意説明書およびその他の説明文書を作成し、必要な場合にはこれを改訂するものとする。作成または改訂された当該文書は、予め倫理委員会の承認を得なければならない。   1. 同意取得の時期、方法およびその内容   　研究責任者または研究分担医師は、対象となる被験者本人に予め倫理委員会で承認を得た同意説明文書を手渡し、十分に説明し、被験者が内容をよく理解したことを確認した上で、本研究への参加について被験者本人の自由意思による同意を文書にて得ることとする。なお、同意書には説明を行った研究責任者または研究分担医師および被験者本人が記名・捺印または署名し、各自日付を記入する。  　なお、同意書の一部を被験者に手渡し、一部を診療録に添付して保存する。  **11 遺伝情報の開示に関する考え方**  　該当なし  **12 研究実施前提供試料等を使用する場合の同意の有無、内容、提供時期、国の倫理指針への適合性**  　該当なし  **13 他の研究機関から試料等又は遺伝情報の提供を受ける場合のインフォームド·コンセントの内容**  　該当なし  **14 試料等又は遺伝情報を外部の機関に提供する場合や研究の一部を委託する場合の匿名化の方法等の事項**  　該当なし  **15 試料等の保存方法及びその必要性**  　該当なし  **16 ヒト細胞·遺伝子·組織バンクに試料等を提供する場合には、バンク名、匿名化の方法等**  　該当なし  **17 遺伝カウンセリングの必要性及びその体制**  　該当なし   1. **資金の調達方法**   　大学院医学研究科消化器内科学講座の研究費により行われ、研究対象者の費用負担はない。 |
| --- |

**（注）別紙「実施計画書に記載すべき事項」に基づき、必要な事項を漏れなく記載すること。該当のない項目は、該当なしと記載すること。**

**説　　明　　文　　書**

| Vbn・  **研究対象者又は代諾者　　　　　　　　様（代諾者の場合研究対象者との関係を記載：　　）**  **１　課題名**  **ナイーブT細胞リッチTリンパ球移入療法の再発・進行癌に対する第I相臨床試験**  **２　実施責任者及び実施担当者の職・氏名**  実施責任者：京都府立医科大学大学院消化器内科 教授・吉川敏一  実施担当者：京都府立医科大学大学院消化器内科 教授・吉川敏一  京都府立医科大学大学院消化器内科 准教授・古倉　聡  京都府立医科大学大学院消化器内科 講師・石川　剛  京都府立医科大学大学院消化器内科 研修員・坂元直行  **３　実施計画の意義及び目的について（医学用語等、実施計画の概要の説明を含む）**  　現在、消化器癌（食道癌、胃癌、結腸癌、直腸癌、膵癌、胆道癌、肝細胞癌）および肺癌に対して、外科的治療、放射線治療、抗がん剤治療が広く行われていますが、これらの治療法によっても根治が期待できない切除不能・再発癌の患者さんに対する治療法として、免疫療法が期待されています。私たちもこれまでに2000回以上の活性化リンパ球療法(CD3-LAK療法)を行い、多くの治療奏功例を経験してきました。また、活性化リンパ球移入に関わる有害事象は一例も経験しておりません。ただ、現在行っているやり方でのCD3-LAK療法を受けていただいた患者さんすべてに十分な治療効果を認めたわけではありません。すなわち、更なる治療効果の改善の必要性を感じています。現在行われているCD3-LAK療法の問題点の一つとして、培養して得られたリンパ球が、患者さんの体内に戻した後の寿命が短いために、リンパ球のがん細胞に対する殺細胞効果が限定的となると考えられています。この問題を克服するために、私たちは、ナイーブTリンパ球を多く含む活性化リンパ球療法の実用化を考えています。ナイーブＴリンパ球とは、未だ抗原に感作されていないＴリンパ球であり、生体内で寿命が長く、体内へ移入後効率よくがん近傍のリンパ節に集まり、そこでがん細胞を攻撃するための能力を獲得することが期待できます。これまでの活性化リンパ球療法でもナイーブＴ細胞は含まれていますがその数は、全体の10-15%位ですが、今回用いるナイーブT細胞リッチTリンパ球移入療法では、移入する細胞の70-80%がナイーブＴ細胞です。本研究では、切除不能・再発消化器癌（食道癌、胃癌、結腸癌、直腸癌、膵癌、胆道癌、肝細胞癌）および肺癌の患者さんに、ナイーブTリンパ球を多く含むTリンパ球移入療法を施行し、その安全性と有効性を科学的に明らかにしたいと考えています。  このような目的の臨床研究ですので、ご協力いただけるようよろしくお願い申し上げます。  **４　実施の方法について**  　本臨床研究に登録された患者さんは、京都府立医科大学消化器内科において、リンパ球培養のための採血を行います。採取された血液から試験細胞（ナイーブTリンパ球を多く含むTリンパ球）の調製を、共同研究機関である百万遍クリニック内の細胞調製室において行います。細胞調製室は、他の患者由来の細胞や外来微生物の混入が起きないよう厳重に管理されており、さらに試験細胞の品質は、培養終了後の品質試験を行うことにより担保しています。すなわち、投与するリンパ球は、一般の治験薬の製造管理および品質管理基準（治験薬ＧＭＰ）に準じて製造・取扱・保管・および管理しています。  　調製された試験細胞は、京都府立医科大学消化器内科において計2回にわけて投与されます。投与間隔は2週間で、2回目の投与4週後までの有害事象の発生の有無や抗腫瘍効果の判定を行います。有害事象の発生しなかった患者さんに対しては、患者さん本人の希望によりさらに４回試験細胞の投与が行われます。  **５　研究対象者等からインフォームド・コンセントを受けるに当たっての説明事項**   1. **実施計画への参加は任意であること。**   　　この計画への協力の同意はあなたの自由意志で決定してください。決して強制いたしません。自由なお気持ちでご判断ください。  **(2)** **実施計画への参加に同意しないことにより不利益な対応を受けないこと。**  この計画への協力の同意をしなくても、あなたは何ら不利益を被ることはありません。  **(3) 同意した場合でも、いつでも文書により不利益を受けることなく撤回することができること**  一旦同意した場合でも、いつでも同意を文書により撤回することができます。その場合、あなたが不利益を受けることは一切ありません。  **(4) 同意が撤回された場合、それまでの研究結果は廃棄されます。**  　　　同意を撤回された場合は、診療記録などがそれ以降は研究目的に用いられることはありません。  **(5) 研究対象者に選ばれた理由**  京都府立医科大学附属病院の消化器内科外来あるいはがん免疫療法外来を受診された20歳以上80歳未満の切除不能・再発消化器癌（食道癌、胃癌、結腸癌、直腸癌、膵癌、胆道癌、肝細胞癌）または肺癌の患者さんであり、重篤な合併症がなく、ナイーブTリンパ球を多く含むTリンパ球移入療法を安全に受けていただけると判断させていただきました。  **(6) 本臨床研究の実施期間**：　承認日　～　平成２２年　３月 ３１日  **(7) 予測される研究結果及び研究対象者等に対して予測される危険や不利益及びその対応**  私たちはこれまでに2000回以上の活性化リンパ球療法(CD3-LAK療法)を行い、多くの治療奏功例を経験してきました。また、活性化リンパ球移入に関わる有害事象は一例も経験しておりません。　ナイーブTリンパ球を多く含むTリンパ球療法は、従来のCD3-LAK療法よりも高い抗腫瘍効果を発揮することが期待されます。有害事象については、患者さん自身の細胞を培養して得ており、重篤な有害事象がおこる可能性は低いものと考えています。出現の可能性がある有害事象として、あなたの細胞を培養する際に使用する培地中に含まれる抗生物質によるアレルギー類似反応や、肺障害および保存用液に用いられる特定生物由来製品に該当するアルブミン製剤投与による感染症の危険などありますが、有害事象が発生した場合には迅速に対応します。  **(8) 研究対象者及び代諾者等の希望により、他の研究対象者等の個人情報の保護や研究の独創性の確保に支障が生じない範囲内で実施計画及び実施方法について資料を入手又は閲覧することができます。**  　すなわち、希望があれば、この実施計画の内容を見ることができます。  **(9)個人情報の保護方法**  本臨床研究に登録していただいた患者さんについては、登録時に決められる登録番号、患者さんのイニシャル、生年月日、カルテ番号を用いて臨床データは保管されます。これらの情報は、研究分担者の消化器内科准教授の古倉　聡が責任を持って管理します。情報管理にはパソコンを使用しますが、このパソコンはネットワークから隔絶された状態で使用および管理されます。本研究では個人情報保護のために最大限の努力を行い、施設、研究事務局間での症例データのやりとりは、紙、電子媒体のいかんにかかわらず、直接手渡しすることを原則とします。本臨床研究では、タカラバイオという会社が外部協力者としてナイーブT細胞リッチTリンパ球の調製技術の提供・助言に限定し、間接的に関与しています。調製されたTリンパ球をあなたに投与した場合の安全性や機能に関する記録は、通常の治験と同様に被験者識別コードを用いることにより個人が特定できないように個人情報を完全に匿名化してから、タカラバイオの担当者が閲覧する可能性があります（被験者識別コードから患者さんを特定する情報については、担当医が厳重に管理します。）また、この治療法をあなたと同じ病気で悩む患者さんに使用いただくために、この臨床研究の結果は、将来、病院だけではなく、この治療法の開発を希望する企業（製薬会社など）にも開示されるかもしれません。他の病院や企業に開示する情報は今回の臨床研究結果に限定したもので、参加していただいたあなたの個人情報（名前など）は記号などに置き換えさせていただき、プライバシー保護は厳守致します。  (10) 研究成果の公表について  あなたの協力によって得られた計画の成果は、提供者本人やその家族の氏名など個人を特定できる情報は一切明らかにされないようにした上で、学会発表や学術雑誌に公に発表されることがあります。  (11) 費用負担に関する事項  本臨床研究に関する診療のうち、Tリンパ球療法にかかる費用については、京都府立医科大学消化器内科の研究費でまかなわれ、患者さんの費用負担はありません。ただし、通常の診療行為の範囲内でおこなわれる投薬、検査に関してはこれまで通り、保険診療として行われます。  **(12) 問い合わせ、苦情等の窓口の連絡先等について**  この実施計画についてのお問い合わせ先は、京都府立医科大学消化器内科において受け付けております。  研究実施担当者：古倉　聡  京都府立医科大学医学部　消化器内科  〒602-8566  京都市上京区河原町広小路上ル梶井町465  電話番号 075-251-5519  **６　説明者の氏名、所属及び捺印並びに説明を行った日時、場所**  **氏名　　　　　　　　　　　　　　　**  **所属**  **日時　　　　　年　　　　月　　　　日**  **場所** |
| --- |

**同　　　意　　　書**

| **実施責任者**  **所属・職　京都府立医科大学大学院　消化器内科・教授**  **氏　　名 吉川敏一様**  **私（氏名）　　　　　　　は、 ナイーブT細胞リッチTリンパ球移入療法の再発・進行癌に対する第I相臨床試験の実施について（説明者）　　　　　　　　　より（日時）　　年　　月　　日、（場所）　　　　　　　　　において説明文書を用いて説明を受け、実施計画の意義、目的、方法、個人情報の保護方法などについて十分理解しましたので計画に参加することを同意いたします。**  **１　説明を受け理解した項目（□の中にご自分でレを付けてください）**  **□　計画の意義及び目的　　　　　　　　　　　　□　計画の実施方法**  **□　計画への参加は任意であること　　　　　　　□　参加に同意した場合でも、随時文書に**  **□　研究対象者等の選定方法　　　　　　　　　　　より撤回できること**  **□　予想される結果、危険性等　　　　　　　　　□　研究対象者等への説明及びインフォー**  **□　個人情報の保護方法　　　　　　　　　　　　　ムド・コンセントの取得方法**  **□　費用負担に関する事項　　　　　　　　　　　□　試料・資料の保管について**      **平成　　年　　月　　日**  **研究対象者**  **氏　　名 印**  **生年月日**  **住　　所**  **電話番号** |
| --- |
