# Supplementary material for: Phase I Clinical Trial of Fibronectin CH296-Stimulated T Cell Therapy in Patients with Advanced Cancer
Source: PLoS One. 2014 Jan 31;9(1):e83786. doi: 10.1371/journal.pone.0083786 (PMC3908868; doi:10.1371/journal.pone.0083786)
Supplement: Flowchart S1 — CONSORT Flow Diagram. (DOC) [file pone.0083786.s002.doc]

**CONSORT 2010 Flow Diagram**

**Allocation**

**Analysis**

**Follow-Up**

**Enrollment**

non-randomized design

Assessed for eligibility (n= 10 )

Excluded (n= 1 )

♦  Not meeting inclusion criteria (n= 1)

♦  Declined to participate (n= 0 )

♦  Other reasons (n= 0 )

Analysed (n= 9 )
♦ Excluded from analysis (give reasons) (n= 0)

Completed follow-up (n=9)

Lost to follow-up (give reasons) (n= 0 )

Discontinued intervention (give reasons) (n= 0 )

Allocated to intervention (n= 9 )

♦ Received allocated intervention (n= 9 )

♦ Did not receive allocated intervention (give reasons) (n= 0 )

Lost to follow-up (give reasons) (n= )

Discontinued intervention (give reasons) (n= )

Allocated to intervention (n= )

♦ Received allocated intervention (n= )

♦ Did not receive allocated intervention (give reasons) (n= )

Analysed (n= )
♦ Excluded from analysis (give reasons) (n= )

Randomized (n= )
