# Supplementary material for: Phase I Clinical Trial of Fibronectin CH296-Stimulated T Cell Therapy in Patients with Advanced Cancer
Source: PLoS One. 2014 Jan 31;9(1):e83786. doi: 10.1371/journal.pone.0083786 (PMC3908868; doi:10.1371/journal.pone.0083786)
Supplement: Checklist S1 — CONSORT checklist. (DOCX) [file pone.0083786.s003.docx]

| **Paper**  **Section/**  **Topic** | **ltem**  **NO** | **Descriptor** | **Reported?** | |
| --- | --- | --- | --- | --- |
|  |  |  | **✓** | **Pg井** |
| **Title and Abstract** | | | | |
| Title and  Abstract | １ | ●　lnformation on how unit were allocated to interventions | ✓ | Abstract |
|  |  | ●　Structured abstract recommended | ✓ | Abstract |
|  |  | ●　lnformation on target population or study sample | ✓ | Abstract |
| **lntroduction** | | | | |
| Background | ２ | ●　Scientific background and explanation of rationale | ✓ | Introduction |
|  |  | ●　Theories used in designing behavioral interventions | ✓ | Introduction |
| **Methods** | | | | |
| Participants | ３ | ● Eligibility criteria for participantsjncluding criteria at different levels in  　　recruitment/sampling plan (e･g･,cities,clinics,subjects) | ✓ | Methods |
|  |  | ● Method of recruitment (e･g･,referral,self-selection)jncluding the  　　sampling method if a systematic sampling plan was implemented | ✓ | Methods |
|  |  | ● Recruitment setting | ✓ | Methods |
|  |  | ● Settings and locations where the data were collected | ✓ | Methods |
| lnterventions | ４ | ●　Details of the interventions intended for each study condition and how  　　and when they were actually administered, specifically including: |  |  |
|  |  | o　Content: what was given？ | ✓ | Methods |
|  |  | o　Delivery method: how was the content given？ | ✓ | Methods |
|  |  | o　Unit of delivery: how were the subjects grouped during delivery? | ✓ | Methods |
|  |  | o　Deliverer: who delivered the intervention? | ✓ | Methods |
|  |  | o　Setting: where was the intervention delivered? | ✓ | Methods |
|  |  | o　Exposure quantity and duration: how many sessions or episodes or  　　events were intended to be delivered? How long were they  　　intended to last? | ✓ | Methods |
|  |  | o　Time span: how long was it intended to take to deliver the  　　intervention to each unit? | ✓ | Methods |
|  |  | o　Activities to increase compliance or adherence (e.g., incentives) | ✓ | Methods |
| Objectives | ５ | ●　Specific objectives and hypotheses | ✓ | Methods |
| Outcomes | ６ | ●　Clearly defined primary and secondary outcome measures | ✓ | Methods |
|  |  | ●　Methods used to collect data and any methods used to enhance the  　　quality of measurements | ✓ | N/A |
|  |  | ●　lnformation on validated instruments such as psychometric and biometric  　　properties | ✓ | Methods |
| Sample Size | ７ | ●　How sample size was determined and, when applicable, explanation of any  　　interim analyses and stopping rules | ✓ | Methods |
| Assignment  Method | ８ | ●　Unit of assignment (the unit being assigned to study condition, e.g.，  　　　individual,group,community) | ✓ | N/A |
|  |  | ●　Method used to assign units to study conditionsjncluding details of any  　　restriction(e.g.，blocking, stratification，minimization) | ✓ | N/A |
|  |  | ●　lnclusion of aspects employed to help minimize potential bias induced due  　　to non-randomization (e.g･,matching) | ✓ | N/A |

TREND Statement Checklist

| Blinding  (masking) | ９ | ● Whether or not participants, those administering the interventions, and  　those assessing the outcomes were blinded to study condition assignment;  　if so,statement regarding how the blinding was accomplished and how it 　　was assessed. | ✓ | N/A |
| --- | --- | --- | --- | --- |
| Unit of Analysis | 10 | ● Description of the smallest unit that is being analyzed to assess  　　　intervention effects (e.g., individual, group, or community) | ✓ | N/A |
|  |  | ●　lf the unit of analysis differs from the unit of assignment, the analytical  　method used to account for this (e.g., adjusting the standard error  　estimates by the design effect or using multilevel analysis) | ✓ | N/A |
| Statistical  Methods | 11 | ●　Statistical methods used to compare study groups for primary methods  　outcome(s), including complex methods of correlated data | ✓ | N/A |
|  |  | ●　Statistical methods used for additional analyses, such as a subgroup  　analyses and adjusted analysis | ✓ | Methods |
|  |  | ●　Methods for imputing missing data, if used | ✓ | N/A |
|  |  | ●　Statistical software or programs used | ✓ | Methods |
| **Results** | | | | |
| Participant flow | 12 | ●　Flow of participants through each stage of the study: enrollment，  　　assignment, allocation, and intervention exposure follow-up, analysis(a  　　diagram is strongly recommended) |  |  |
|  |  | o　Enrollment: the numbers of participants screened for eligibility，  　　　found to be eligible or not eligible, declined to be enrolled, and  　　　enrolled in the study | ✓ | Results |
|  |  | o　Assignment: the numbers of participants assigned to a study  　　condition | ✓ | Results |
|  |  | o　AIlocation and intervention exposure: the number of participants  　　assigned to each study condition and the number of participants  　　who received each intervention | ✓ | N/A |
|  |  | o　Follow-up: the number of participants who completed the follow-  　　up or did not complete the follow-up (i.e., lost to follow-up),by  　　study condition | ✓ | Results |
|  |  | o　Analysis: the number of participants included in or excluded from  　　the main analysis, by study condition | ✓ | Results |
|  |  | ●　Description of protocol deviations from study as planned, along with  　　reaSOnS | ✓ | N/A |
| Recruitment | 13 | ●　Dates defining the periods of recruitment and follow-up | ✓ | Results |
| Baseline Data | 14 | ●　Baseline demographic and clinical characteristics of participants in each  　study condition | ✓ | Results  Table 1 |
|  |  | ●　Baseline characteristics for each study condition relevant to specific  　disease prevention research | ✓ | N/A |
|  |  | ●　Baseline comparisons of those lost to follow-up and those retained, overall  　and by study condition | ✓ | N/A |
|  |  | ●　Comparison between study population at baseline and target population  　of interest | ✓ | N/A |
| Baseline  equivalence | 15 | ●　Data on study group equivalence at baseline and statistical methods used  　　to control for baseline differences | ✓ | N/A |

TREND Statement Checklist

| Numbers  analyzed | 16 | ●　Number of participants (denominator)induded in each analysis for each  　study condition, particularly when the denominators change for different  　outcomes; statement of the results in absolute numbers when feasible | ✓ | N/A |
| --- | --- | --- | --- | --- |
|  |  | ●　lndication of whether the analysis strategy was ″intention to treat″ orjf  　not,description of how non-compliers were treated in the analyses | ✓ | N/A |
| Outcomes and  estimation | 17 | ●　For each primary and secondary outcome, a summary of results for each  　estimation study condition, and the estimated effect size and a confidence  　interval to indicate the precision | ✓ | Results  Table3  Table4 |
|  |  | ●　lnclusion of null and negative findings | ✓ | N/A |
|  |  | ●　lnclusion of results from testing pre-specified causal pathways through  　which the intervention was intended to operate, if any | ✓ | N/A |
| Ancillary  analyses | 18 | ●　Summary of other analyses performed, including subgroup or restricted  　analyses, indicating which are pre-specified or exploratory | ✓ | Results |
| Adverse events | 19 | ●　Summary of all important adverse events or unintended effects in each  　study condition (induding summary measures, effect size estimates, and  　confidence intervals) | ✓ | Results |
| **DISCUSSION** | | | | |
| lnterpretation | 20 | ●　lnterpretation of the results, taking into account study hypotheses，  　sources of potential biasjmprecision of measures, multiplicative analyses，  　and other limitations or weaknesses of the study | ✓ | Discussion |
|  |  | ●　Discussion of results taking into account the mechanism by which the  　intervention was intended to work (causal pathways) or alternative  　mechanisms or explanations | ✓ | Discussion |
|  |  | ●　Discussion of the success of and barriers to implementing the intervention，  　　fidelity of implementation | ✓ | Discussion |
|  |  | ●　Discussion of research, programmatic, or policy implications | ✓ | Discussion |
| Generalizability | 21 | ●　Generalizability(externalvalidity) of the trial findings, taking into account  　the study population, the characteristics of the intervention，length of  　follow-upjncentives,compliance rates, specific sites/settings involved in  　the study, and other contextual issues | ✓ | Discussion |
| Overall  Evidence | 22 | ●　Genera目nterpretation of the results in the context of current evidence  　　　and current theory | ✓ | Discussion |

TREND Statement Checklist

From: Des Jarlais, D.C.，Lyles,C.，Crepaz，N.,＆the Trend Group (2004). Improving the reporting quality of

nonrandomized evaluations of behavioral and public health interventions: The TREND statement. *American Journal of*

*Public Health*, 94,361-366.For more information, visit: http://www.cdc.ov.trendstatement
